# Quality Visualization of Microarray Datasets Using Circos

**DOI:** 10.3390/microarrays1020084

**Published:** 2012-08-07

**Authors:** Martin Koch, Michael Wiese

**Affiliations:** Pharmaceutical Institute, Rheinische Friedrich Wilhelms University Bonn, An der Immenburg 4, Bonn 53121, Germany; Email: martin.koch@uni-bonn.de

**Keywords:** circos, yaqcaffy, quality monitoring

## Abstract

Quality control and normalization is considered the most important step in the analysis of microarray data. At present there are various methods available for quality assessments of microarray datasets. However there seems to be no standard visualization routine, which also depicts individual microarray quality. Here we present a convenient method for visualizing the results of standard quality control tests using Circos plots. In these plots various quality measurements are drawn in a circular fashion, thus allowing for visualization of the quality and all outliers of each distinct array within a microarray dataset. The proposed method is intended for use with the Affymetrix Human Genome platform (*i.e.*, GPL 96, GPL570 and GPL571). Circos quality measurement plots are a convenient way for the initial quality estimate of Affymetrix datasets that are stored in publicly available databases.

## 1. Introduction

### 1.1. Microarray Raw Data and Quality Control

Microarray technology has successfully made the transition from a specialized method to a common method that is widely adopted in biological research. However, marginal overlap in gene expression profiling studies [1] and discussions about analysis approaches for identifying differentially expressed genes have prompted a microarray quality initiative. The microarray quality control project (MAQC) aims to estimate the accuracy of the technology [2] and examines in a second phase analysis methods and obtained biological models [3]. In general, the aim of the MAQC-II study was to resolve the concerns about the reproducibility and the generalization capability of microarray analysis results, which may stem from:

(i) a lack of information about the analysis protocol.
(ii) choosing different normalization methods.
(iii) the use of defective analytical methods.

It was found that reproducibility seems to rely greatly on the availability and completeness of the documentation of the analysis process; however it is just as essential to start the analysis with high quality data. Therefore, prior to the analysis of the raw data, the quality of the dataset needs to be assessed. An important goal of quality assessment is detection of outliers. However, currently there is no common quality measure to estimate the soundness of a microarray dataset, although there are a variety of methods available for the analysis of microarray quality in the Bioconductor project [4]. A remedy to this situation is given by post-normalization quality assessment, which detects systematically wrong appliance of normalization. It was earlier suggested that a quality check should be performed before the actual analysis as well, thus preventing the attempt of normalizing erroneous data in the first place [5]. 

In general normalization methods are specifically designed either for dual channel microarray datasets [6,7,8,9,10] or single channel microarray datasets [11,12]. However, there are also manufacturer dependent normalization methods available for the different flavors of microarray data [12,13,14,15,16,17,18,19,20,21,22]. 

### 1.2. Aim of the Present Study

Here we present a convenient visualization approach for assessment of individual microarray quality in large data sets using Circos. We have applied state of the art Bioconductor packages, to obtain quality control values. To condense the quality control analysis results, only outliers in the assessed quality measurements are highlighted, *i.e*., via red dots, denoting absence or deviations of probes in standard technical controls. Additionally RNA degradation assessment is presented in tiles using a gradient from blue to red for depicting potential outlier probes. Finally a principal component plot shows the actual array as a red dot and the other arrays in blue, thus facilitating the detection of potential outlier candidates. Presenting the analysis results in a circular format might be a suitable and convenient way for the initial quality estimate of datasets that are stored in publicly available databases. R code and a demo are publicly available at: https://github.com/buzzmak/circos-arrayQC. 

## 2. Experimental Section

### 2.1. Publicly Available Microarray Studies in GEO

Microarray raw data of publicly available studies was obtained from Gene Expression Omnibus (GEO, [23]). Table 1 lists all studies, which were evaluated for array quality; note that all studies comprise Affymetrix Human Genome U133A arrays and became available recently, *i.e*., 2011, except for GSE9936 [24] which was submitted to GEO in 2008.

**Table 1:** The following studies are publicly available as microarray raw data in the Gene Expression Omnibus (GEO) database.

GEO ID	Samples	Reference	Published on
GSE9801	6	[25]	7 November 2011
GSE32700	46	[26]	7 October 2011
GSE9936	105	[24]	7 February 2008

### 2.2. Data Processing, Normalization and Principal Component Analysis

Microarray data handling was performed in R (2.15.0), using the latest version of Bioconductor [4]. Microarray raw data was obtained using the *GEOquery* package [27] and batch processed using the *affy* package [28]. Additionally we used *affy* to calculate potential candidate arrays for RNA degradation. We performed quality control tests using the *yaqcaffy* package, which provides routines that are dedicated to Affymetrix arrays. We calculated all outliers in average background and average noise. Additionally we estimated outliers in both house-keeping probes (*i.e.*, β-Actin and GADPH), as well as outliers in the internal spike-in probe calls and poly-A controls. 

Finally all microarrays were processed using quantile normalization of the *RMA* package without background adjustment [18]. Subsequently principal component analysis was performed using the *pcaMethods* package [29]. The scores of the mean centred first principal component were obtained for visualization.

### 2.3. Data Visualization Using Circos

All quality control analysis results were summarized using R and then all dedicated Circos input files were generated by use of the proposed method (R code and a demo are publicly available at: http://github.com/buzzmak/circos-arrayQC). 

Inside an R command shell the proposed method can easily be executed, calling the following routine:

***> writeCircos.files(data, celNames, workdir, fileName, pathToCircos)***

Where ***data*** presents an *affyBatch* object containing the raw data, ***celNames*** denotes all Affymetrix cel-file names used in the analysis, ***workdir*** is the path to the current directory, ***filename*** names the resulting Circos QC plot and ***pathToCircos*** leads to the home directory of Circos. 

Circos [30] version 0.6 and strawberry Perl (http://strawberryperl.com/) version 5.1.16 was used to generate all circular quality plots. Note that the Circos method needs to be called by the Perl interpreter, *i.e*.,

***> perl bin/circos –conf C:\Path\to\where\circosFiles\are\located\circosQCconfig.txt***

## 3. Results and Discussion

### 3.1. Quality Assessment and Normalization of Diverse Studies Available in GEO

**Figure 1:**
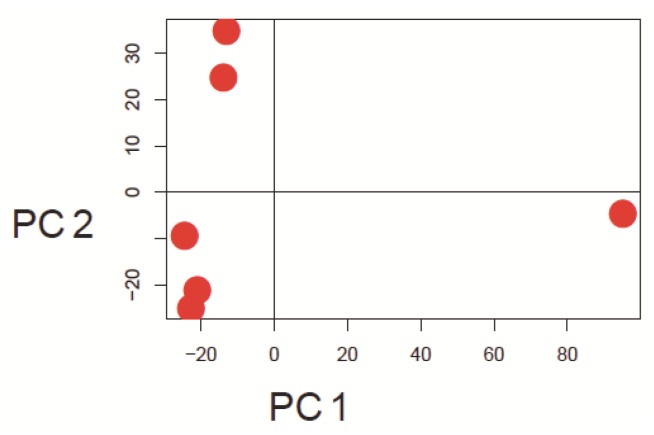
Principal component analysis was performed using the *pcaMethods* package. The figure depicts the first *versus* the second principal component in a scatter plot. There are two clusters and one potential outlier to the right in dataset GSE9801. The first PC explains 67% and the second PC explains 19% of the variance of the data.

**Figure 2:**
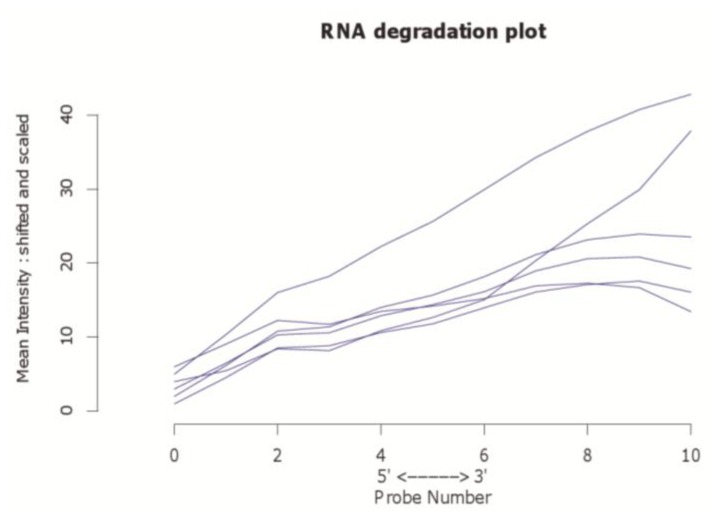
The *affy* package enables the examination of potential RNA degradation probes, *i.e.*, eleven control probes which can reveal a potential fragmentation with high significance. Here we depict potential RNA degradation of arrays in dataset GSE9801. Note that the topmost blue line represents an outlier.

**Figure 3:**
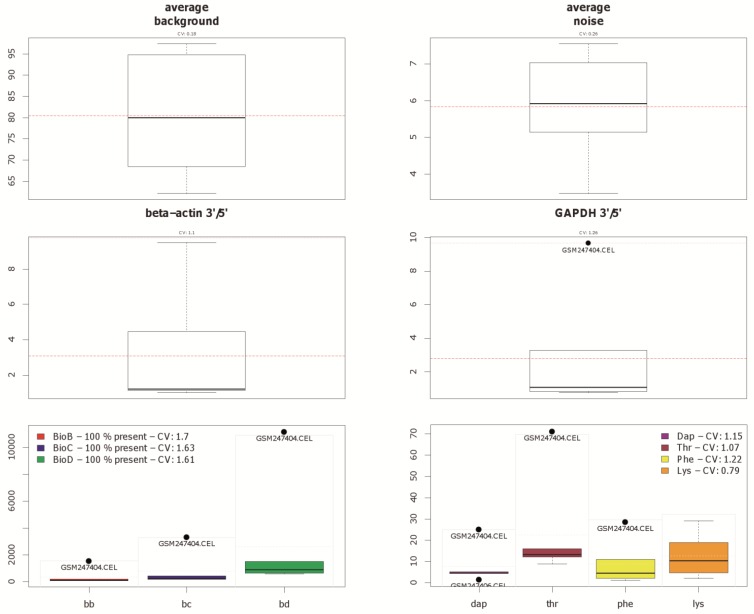
Different quality measurements, which are available in the Bioconductor package yaqcaffy, shown for the example of dataset GSE9801. The first row contains two box-plots, which denote the average background and noise. The second row contains another two box-plots, showing the expression status of so called housekeeping genes. The third row contains box-plots of the Affymetrix specific spike-in and poly-A controls. Note that array GSM247404 is highlighted as potential outlier by black dots. Array GSM247406 is also flagged as outlier in the dap poly-A control, as seen at the bottom right.

Since currently the majority of microarray datasets in GEO are based on Affymetrix arrays, we focused our investigation on Human Genome U133A arrays (GPL96 and GPL570) from this platform. Three representative example datasets varying mainly in the number of comprised arrays were selected to show the visualization capability of our approach (Table 1). The principal approach is introduced by using arrays from the study GSE9801. The principal component analysis results, which are made with the *pcaMethods* package [29], are depicted in Figure 1 for this dataset. Here the first principal component is plotted against the second principal component. Two clusters and one potential outlier array can be seen. In Figure 2, a plot of potential RNA degradation is presented, generated with the *affy* package [28]. One array, which represents the topmost line, is probably an outlier. The *yaqcaffy* (http://www.bioconductor.org/packages/release/bioc/html/yaqcaffy.html) package in Bioconductor provides several quality assessment methods for Affymetrix arrays. In Figure 3 there is a quality analysis plot of study GSE9801 made with *yaqcaffy*. All quality control measurements are demonstrated and the suggested cut-off values are displayed as well. The box-plot showing the GADPH present calls reveals that array GSM247404 is probably an outlier. In addition, the same array is shown also in the Affymetrix specific spike-in and poly-A control plots and therefore most probably presents an outlier. Also there is array GSM247406, which has low values in the dap poly-A control.

### 3.2. Visualization by Use of Circos

**Figure 4:**
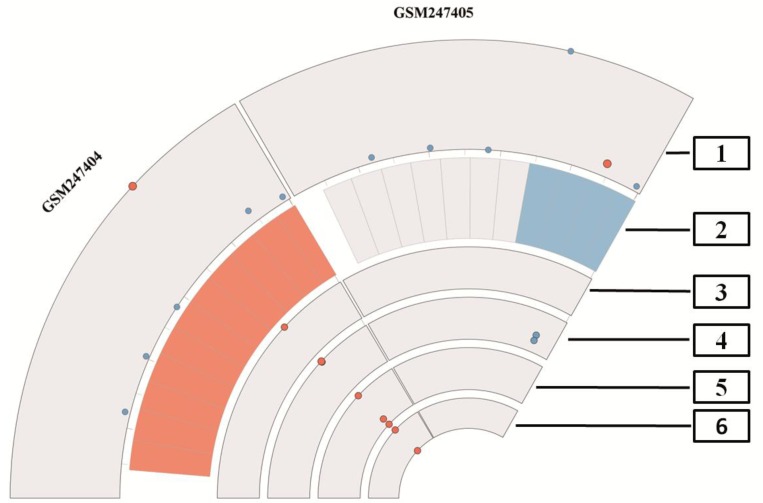
Plot of the different quality measurements, which are shown in Figure 1, Figure 2, Figure 3 of the dataset GSE9801 combined with Circos. The outer rim depicts the first principal component, which shows the actual array in red. The second rim displays the result of RNA degradation assessment. Here the red tiles present high values and the blue tiles denote low values of degradation. In addition, white tiles show medium degradation. The next four rims present outliers based on different quality control levels, first average noise and background, second outliers in the level of housekeeping genes and lastly two rims, which are showing outliers in the control spike-in probes. The index in the figure denotes: 1 = first principal component. Note that the actual array is presented as a red dot for better visualisation. 2 = RNA degradation, 3 = background and noise, 4 = β-Actin and GADPH, 5 = internal spike-in probe calls, 6 = poly-A controls. Note that array GSM247404 presents outliers on each level, even when the scores of the first principal component clusters are apart. In contrast to that, the array GSM247405 depicts inconspicuous values for almost all quality measurements, even GADPH and β-Actin shown on the fourth level in blue appear to be of good quality.

**Figure 5:**
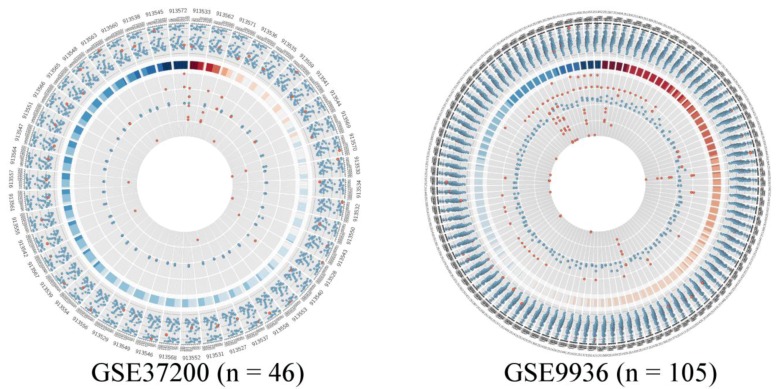
Quality measurement plots of the two datasets listed in Table 1, visualized by Circos, investigating the visualization limit of the proposed method. The plot on the left shows the results of the quality assessment from 46 arrays. The plot on the right summarizes all quality measurements for more than 100 arrays. In this plot one can only trace the quality of the whole dataset; as assessing the quality of single arrays without magnifying is difficult. Therefore we suggest inspecting the same plot in scalable vector format (*i.e*., svg), as provided in Figure S1 of the supplement.

In Figure 4 we present an overview of the proposed quality measurement plot, using Circos. The plot combines all previously mentioned quality measurement methods and shows also quality measurements of individual arrays. Here we depict only two arrays, for explanatory purposes. Figure 5 depicts all arrays of a dataset in a circular view. Also we wished to condense quality information almost to the presence of outliers, in this way focusing only on erroneous outlier arrays. The first rim depicts the first principal component and highlights the actual array as a red dot, which is slightly bigger than the blue dots that represent the other arrays. Here arrays having similar principal component scores are clustered together, whereas arrays having different principal component scores are located apart.

In the next rim potential RNA degradation is depicted. A low overall quality is shown as red tile, while in the case when no degradation was measured the tiles are blue. Medium RNA degradation is depicted in white; these probes are in the range of the tolerance limits. For a better visual inspection of the whole dataset, all arrays are grouped together according to the RNA degradation measure. This way, arrays of similar RNA quality are typically positioned aside in the plot. In the third rim we find red dots if the average background or average noise measurements is off scale. The following three rims are dedicated to several affymetrix specific technical quality control probes and are introduced as follows. The fourth rim comprises the house-keeping genes β-actin and GADPH in blue and flagged in red in case the probes are off scale, *i.e*., induced more than threefold, these probes will be depicted in red. The fifth rim presents potential outliers of the internal probe calls, *i.e.*, three probes as depicted in Figure 3 on the bottom left part. The inner ring reveals outliers in poly-A control probes, *i.e.*, maximum of four probes, as depicted in the Figure 3 on the bottom right part. In Figure 4 we present only two arrays from dataset GSE9801, there is one array of low (GSM247404) and another array (GSM247405) of good quality. Array GSM247404 can easily be identified as a potential outlier on all measured quality scales. All technical spike-in probes in rim five are problematic and 50 percent of the other technical controls are outliers as well. The other array (GSM247405) in this dataset however is of good quality, since it shows blue tiles in the RNA degradation plot and no outliers in the technical controls.

We wanted to assess the visual capability of our approach and therefore we generated Circos quality plots for two microarray studies as shown in Figure 5. These studies are listed in Table 1 in detail. The left plot in Figure 5 presents all arrays in study GSE37200 comprising 46 arrays and the right plot shows study GSE9936, which contains 105 arrays. In the dataset GSE32700, there are only three arrays of questionable quality. Two of them present outliers, since RNA degradation is detected and there are outliers in average background or noise levels. In addition to this, on the second innermost ring all three spike-in control probes are potential outliers. The majority of the arrays in the dataset are of good quality, there are only occasionally some outliers, mostly in the poly-A controls. 

The dataset GSE9936 has several RNA degradation candidate arrays, however only one of these arrays has also additional outliers on all other scales. The other RNA degradation candidates have mostly outliers in the average background intensities. The arrays, where RNA degradation is not problematic show off limit values in average background and noise levels instead. Additionally these arrays have off-scale measurements in both house-keeping control genes and also several absent probes and outliers in the technical spike-in and poly-A controls. In total we can count seven arrays, where quality measurements would imply outlier candidates. This underlines the fact that it might be important to access a plurality of quality measurement methods, to gain outlier arrays with greater confidence. In summary our method suits the majority of the dataset series based on GPL96 and GPL570, since most of these comprise less than 100 arrays. However, we find also that datasets containing more than 100 arrays are not reasonably well pictured using our method. Nonetheless, our method is applicable especially for use in web-resources, since Circos produces scalable vector graphic images in which the area of interest can be easily magnified.

## 4. Discussion

The NCBI resource GEO contains at present over 10,000 platforms, of which 1,895 comprise human genomic sequences. Among all samples in GEO (776,566) there are 108,119 samples (13.9%), which are based on the Affymetrix Human Genome platform (*i.e.*, GPL 96, GPL570 and GPL571). Over 13,000 samples are based on the Agilent 4x44k platform (0.94%) and there are 3550 samples based on the Illumina human-6 2.0 expression beadchip platform (0.45%). The proposed Circos method supports quality estimation for the majority of microarray-based experiments, which are based on the Affymetrix platform. However, there are quality-reporting methods for other platforms *i.e.*, beadArray [31] for Illumina data and a proprietary method for Agilent arrays. A standard visualization routine, which also depicts individual microarray quality, could allow for an initial quality estimation of a microarray dataset. In the proposed Circos quality plots we find a promising approach providing quick quality assessment, which could in future be generalized to other microarray platforms as well.

## 5. Conclusion

Quality control is the most important initial step in the analysis of microarray data. However, until now there has been no standard visualization routine to access individual microarray quality control values of large datasets. Here we present a convenient method for accessing the results of standard quality control test results using Circos. Currently the method works only for Microarray datasets, which are based on the Affymetrix Human Genome platform (*i.e.*, GPL 96, GPL570 and GPL571). In future, this method could be adopted for quality estimation of genomic high throughput data.

## Appendix

**Figure S1:**
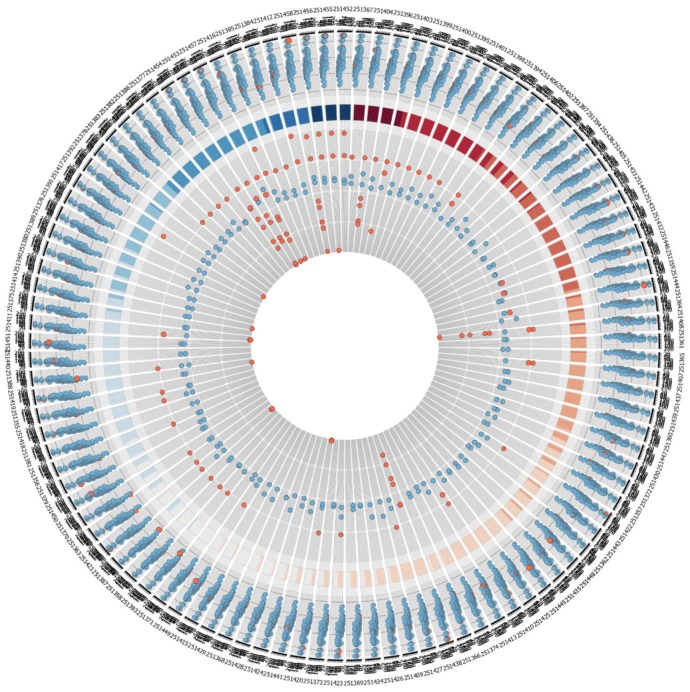
Larger version of the quality measurement plots of dataset GSE9936 as visualized by Circos.
